# Qualitative Analysis of the Literature on Distraction-Based Techniques for Early-Onset Scoliosis

**DOI:** 10.7759/cureus.99132

**Published:** 2025-12-13

**Authors:** Juan Máximo Molina-Linde, Ana María Carlos-Gil, Maria Piedad Rosario-Lozano, Juan Ramón Lacalle-Remigio

**Affiliations:** 1 Health Technology Assessment Area (AETSA), Andalusian Public Foundation Progress and Health (FPS), Seville, ESP; 2 Health Technology Assessment Area (AETSA), General Secretariat of Public Health and Health Research and Development and Innovation, Ministry of Health and Consumer Affairs, Seville, ESP; 3 Preventive Medicine and Public Health, University of Seville, Seville, ESP

**Keywords:** distraction-based techniques, early-onset scoliosis, nvivo, qualitative analysis, systematic review, word cloud

## Abstract

Early-onset scoliosis (EOS) presents significant clinical challenges due to its impact on growth and pulmonary development. Because the available evidence is heterogeneous and high-quality comparative trials are scarce, we incorporated an exploratory qualitative content analysis to complement the systematic review. Fifty documents, including comparative studies, systematic reviews, a health technology assessment report, and economic evaluations, were analyzed by using NVivo qualitative analysis software (QSR International, Melbourne, Australia) and categorized into six thematic groups. These included studies evaluating the three main distraction-based techniques, traditional growing rods, magnetically controlled growing rods, and Vertical Expandable Prosthetic Titanium Rib (VEPTR). Word-cloud visualizations showed that most studies consistently emphasized patients, surgical techniques, deformity correction, and growth-related aspects, represented by concepts associated with curve correction and spinal height gain, such as Cobb angle and T1-S1 height. Follow-up and procedural aspects also appeared frequently, whereas economic evaluations focused on costs and resource use. In contrast, clinically important areas such as pulmonary function, health-related quality of life, and long-term outcomes were infrequently addressed across categories, revealing major evidence gaps. This qualitative approach highlights the dimensions most commonly explored in the literature, identifies underrepresented outcomes relevant to clinical decision-making, and underscores the need for future research that integrates functional and patient-centered measures to support comprehensive care planning in EOS.

## Introduction and background

Systematic reviews are widely regarded as the gold standard for evidence synthesis in the health sciences, as they integrate data from primary studies and formally assess the certainty of the evidence using standardized frameworks [1,2]. However, in highly heterogeneous fields such as the management of early-onset scoliosis (EOS) with distraction-based growth-friendly techniques, the diversity of surgical procedures, outcome measures, follow-up durations, and study designs often limits the feasibility and robustness of traditional meta-analyses [3].

These methodological constraints may restrict the ability to draw comprehensive or reliable conclusions, particularly when high-quality comparative trials are scarce. In this context, qualitative content analysis provides a complementary perspective by identifying thematic patterns, recurrent concepts, and areas of emphasis that may remain obscured in quantitative synthesis alone [4,5]. Software tools such as NVivo (QSR International, Melbourne, Australia) further support this approach by enabling structured coding of texts and providing visual resources, such as word clouds, that intuitively illustrate the relative frequency of terms and help highlight research trends or potential evidence gaps.

EOS, defined as scoliosis greater than 10° diagnosed in children younger than 10 years of age, encompasses a broad and heterogeneous group of conditions that arise from diverse etiologies, including idiopathic, neuromuscular, congenital, and syndromic forms, and may involve abnormalities of the spine, thorax, or both [6]. These deformities can substantially impair thoracic growth and pulmonary development, leading to reduced respiratory capacity, altered chest wall mechanics, and variable functional limitations. In response to these clinical challenges, several distraction-based growth-friendly techniques, such as traditional growing rods (TGR), magnetically controlled growing rods (MCGR), and the vertical expandable prosthetic titanium rib (VEPTR), have been developed to control deformity progression while preserving spinal and thoracic growth [6]. However, these procedures differ markedly in surgical strategies, lengthening protocols, implant biomechanics, and complication profiles, contributing to considerable variability in reported outcomes across the literature [7].

Given the clinical and methodological challenges described, this study aims to evaluate the usefulness of qualitative analysis with NVivo as a complement to systematic review methodology, offering a broader and more nuanced understanding of the existing evidence and helping to identify research gaps that may guide future investigations.

## Review

Materials and methods

Study Design

This was a secondary study based on a systematic review of the literature combined with a qualitative content analysis of the included texts. This review followed the recommendations of the Preferred Reporting Items for Systematic Reviews and Meta-Analyses (PRISMA) 2020 statement [2], including structured reporting of the search strategy, eligibility criteria, study selection process, data extraction procedures, and the use of a PRISMA flow diagram. It aimed to evaluate the effectiveness and safety of distraction-based growth-friendly techniques in patients with EOS, while also exploring the added value of qualitative synthesis as a complement to quantitative evidence.

Search Strategy and Study Selection

A comprehensive bibliographic search was performed in MEDLINE (via PubMed and Ovid), EMBASE, the Cochrane Library, Web of Science (WOS), Trip Medical Database, the International Network of Agencies for Health Technology Assessment (INAHTA), the National Institute for Health and Care Excellence (NICE), the Canadian Agency for Drugs and Technologies in Health (CADTH), and the Spanish Network of Health Technology Assessment Agencies (RedETS). PubMed and Ovid MEDLINE were both searched because they index the complete MEDLINE database through different interfaces, with PubMed also providing in-process and ahead-of-print records. Clinical trial registries were also consulted, including ClinicalTrials.gov, the International Standard Randomised Controlled Trial Number registry (ISRCTN), the International Clinical Trials Registry Platform (ICTRP), the EU Clinical Trials Register, and the Cochrane Central Register of Controlled Trials (CENTRAL).

Whenever possible, the search strategy combined controlled vocabulary (e.g., MeSH, Emtree) with free-text terms related to EOS and distraction-based growth-friendly techniques (growing rods, VEPTR, and MCGR). In databases that do not support controlled vocabulary, only free-text terms were used. The search covered the period from January 2012 to September 2023.

To identify additional sources, Google Scholar was searched to capture gray literature, and the reference lists of all included studies were manually screened through handsearching, with potentially relevant citations recorded in Microsoft Excel (Microsoft Corporation, Redmond, Washington). INAHTA, NICE, CADTH, and RedETS were used exclusively to identify secondary evidence, such as health technology assessment (HTA) reports.

Inclusion and Exclusion Criteria

Eligible studies included systematic reviews, meta-analyses, and comparative primary studies evaluating distraction-based growth-friendly techniques (specifically the VEPTR, TGR, and MCGR) in pediatric patients with EOS. Only quantitative studies with at least ten patients and published in English, Spanish, Italian, or French were included to ensure robustness and avoid anecdotal evidence.

Exclusion criteria comprised narrative reviews, editorials, letters, notes, conference abstracts without original data, preclinical animal or ex vivo studies, and publications limited to technical descriptions without clinical evaluation of effectiveness or safety. Single case reports and very small case series were also excluded, as they provide anecdotal information and cannot be meaningfully synthesized with larger comparative studies.

Qualitative Content Analysis

Full-text articles of the included studies were processed using NVivo 12 Plus software (QSR International) [8]. Coding was independently performed by two reviewers, and discrepancies were resolved by consensus to increase transparency and reliability. The documents were then classified into analytical categories, predefined on the basis of the initial grouping of study types to organize the corpus into logical groups and facilitate comparative analysis.

An exploratory qualitative content analysis was then conducted. This process involved the removal of stop words to eliminate highly frequent but non-informative terms that could distort the relative weight of meaningful concepts [4,5]. Certain discipline-specific terms (such as study) were intentionally retained, as they are inherent to the structure of scientific articles and removing them could omit relevant contextual information. The analysis also included the calculation of absolute word frequency within each study group. Word clouds were generated and understood not only as visual tools but as graphical representations of term frequency that help identify dominant concepts within each thematic category. Word clouds were selected as the primary visual output because they offer an intuitive and concise representation of term prominence, consistent with the exploratory aims of this qualitative analysis. In this exploratory analysis, terms were processed in their original form without automatic stemming or lemmatization; therefore, singular/plural or verb-noun variants (e.g., rod/rods, growth/growing) were retained as they appeared in the source texts. This approach preserves fidelity to the original wording while acknowledging that closely related terms may not be merged into a single lexical root. These visual representations were used to complement the quantitative synthesis by identifying thematic patterns and providing an overview of the predominant concepts in the literature on distraction-based growth-friendly techniques.

Results

Study Selection Process

**Figure 1:**
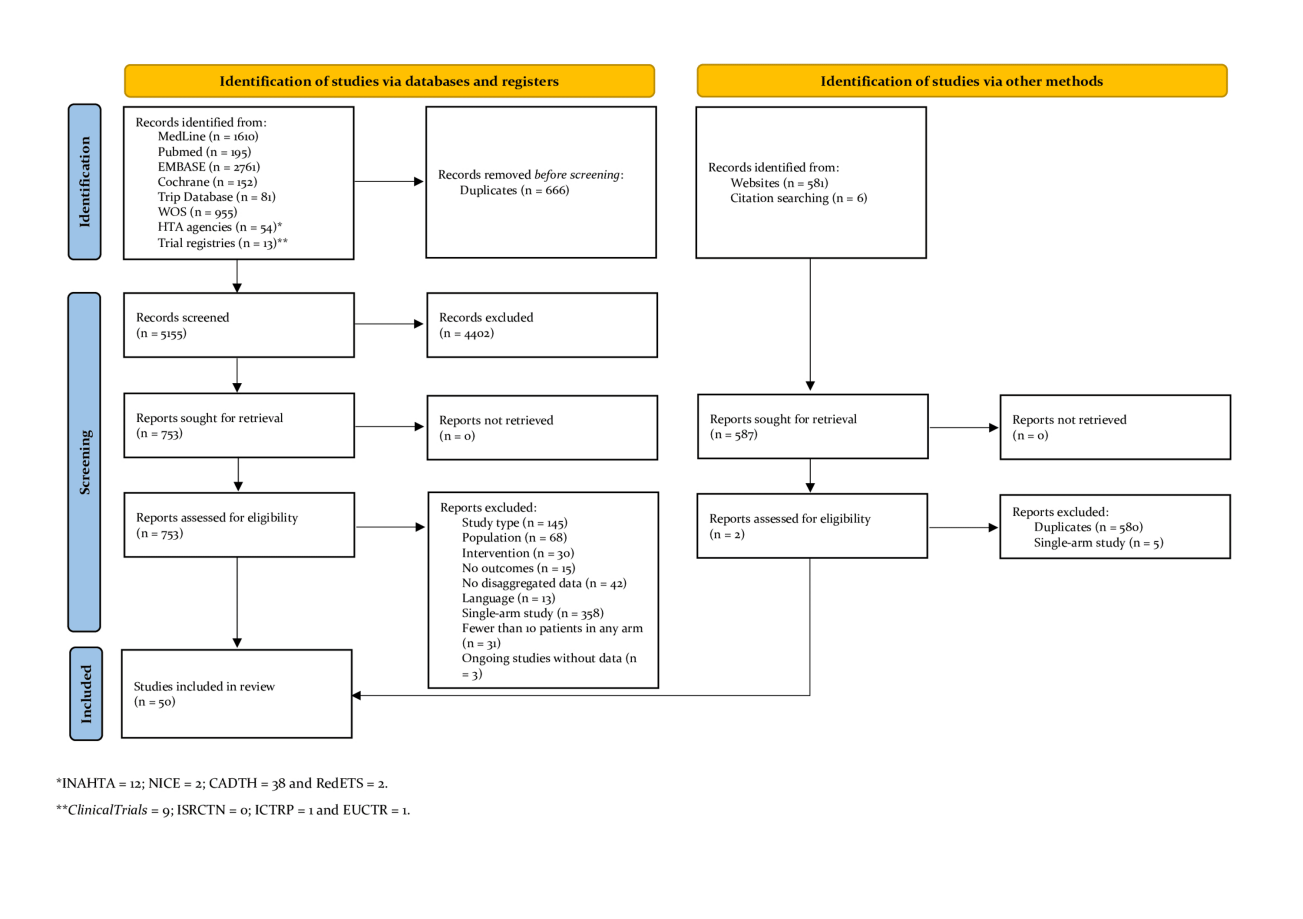
PRISMA flow diagram of the study selection process PRISMA: Preferred Reporting Items for Systematic Reviews and Meta-Analyses.

The search retrieved a total of 6,408 records. After removing duplicates and applying the pre-established eligibility criteria, 50 studies were included in the qualitative and quantitative synthesis. These comprised 29 comparative primary studies [9-37], 12 systematic reviews [38-49], one HTA report [50], and eight original economic evaluations [51-58], one of which was embedded within a systematic review [38]. The complete study selection process is illustrated in the PRISMA flow diagram (Figure 1).

Analyzed Corpus

The full texts included in the review were organized into six analytical categories for qualitative analysis with NVivo 12 Plus. These categories comprised systematic reviews and the HTA report, studies jointly comparing TGR, MCGR, and the VEPTR, comparisons between TGR and VEPTR, comparisons between TGR and magnetically controlled growing rods, comparisons between MCGR and VEPTR, and economic studies evaluating costs associated with distraction-based techniques. This categorization enabled the identification of thematic patterns across different types of evidence.

Thematic Patterns Identified

**Figure 2:**
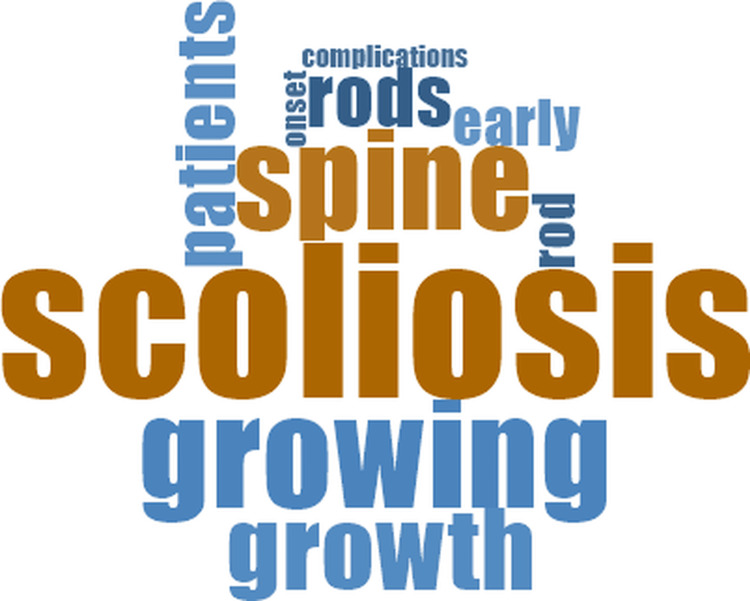
Word cloud of the full-text content from systematic reviews and the health technology assessment report

The qualitative content analysis showed that the different analytical categories contained recurrent terms. In systematic reviews and in the HTA report, the word-cloud visualizations displayed terms such as *scoliosis*, *spine*, *patients*, *growth*, and *complications* (Figure 2).

**Figure 3:**
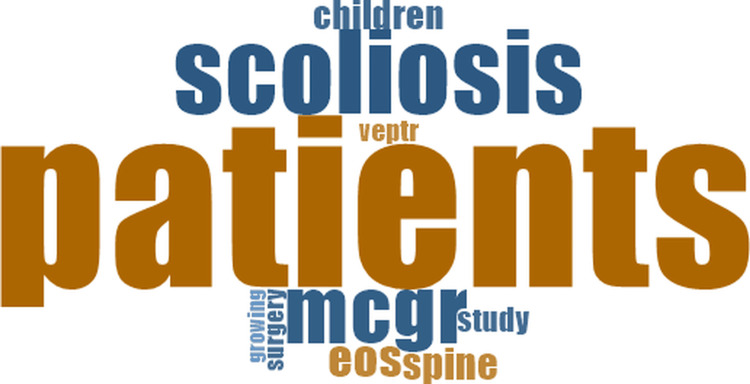
Word cloud of studies comparing the three main techniques: traditional growing rods (TGR), magnetically controlled growing rods (MCGR), and vertical expandable prosthetic titanium rib (VEPTR)

In studies jointly comparing the three distraction-based techniques, the visualizations frequently included terms such as *patients*, *scoliosis*, and the specific names of the devices evaluated (Figure 3).

**Figure 4:**
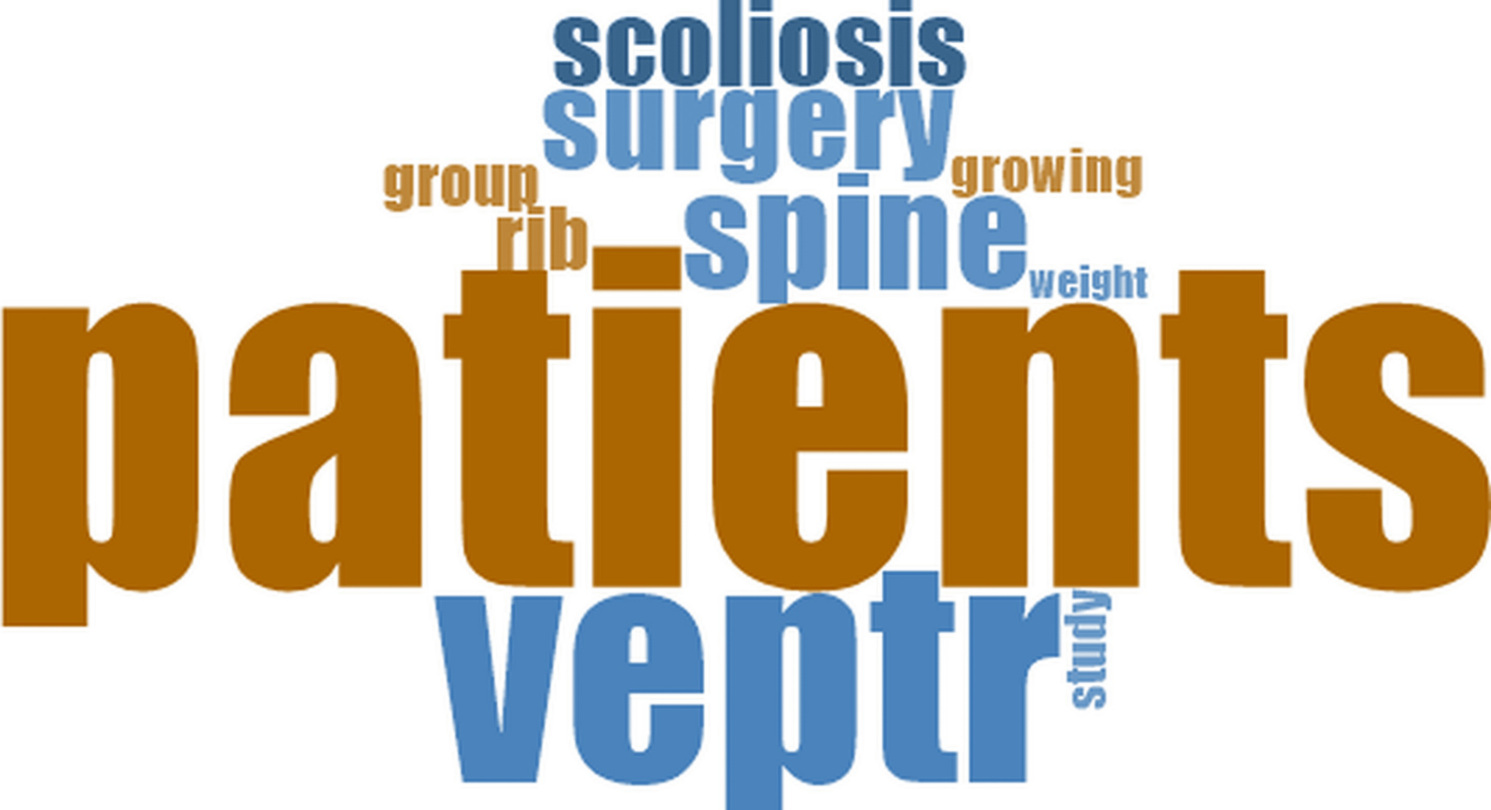
Word cloud from comparative studies between traditional growing rods (TGR) and vertical expandable prosthetic titanium rib (VEPTR) devices

**Figure 5:**
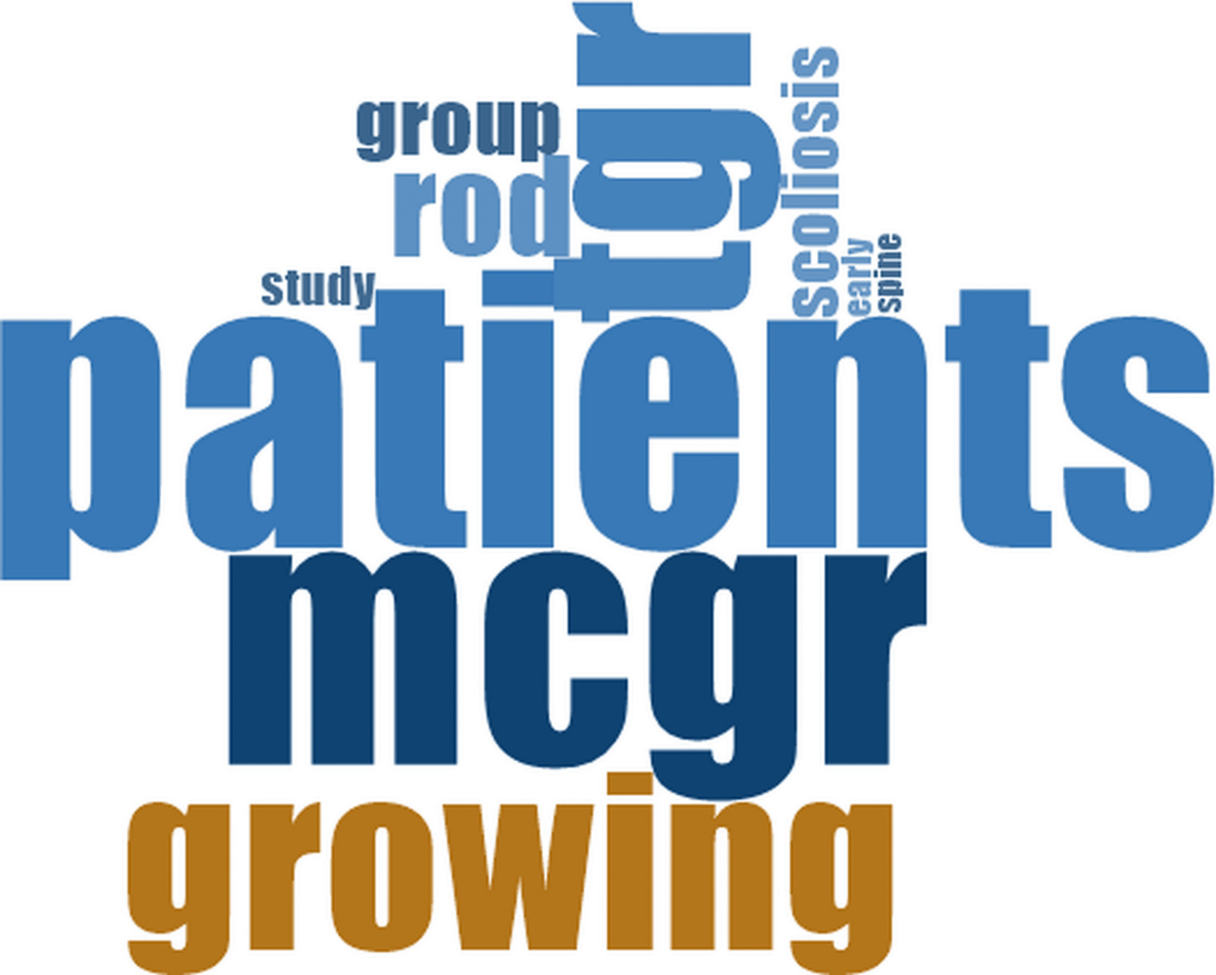
Word cloud from comparative studies between traditional growing rods (TGR) and magnetically controlled growing rods (MCGR)

**Figure 6:**
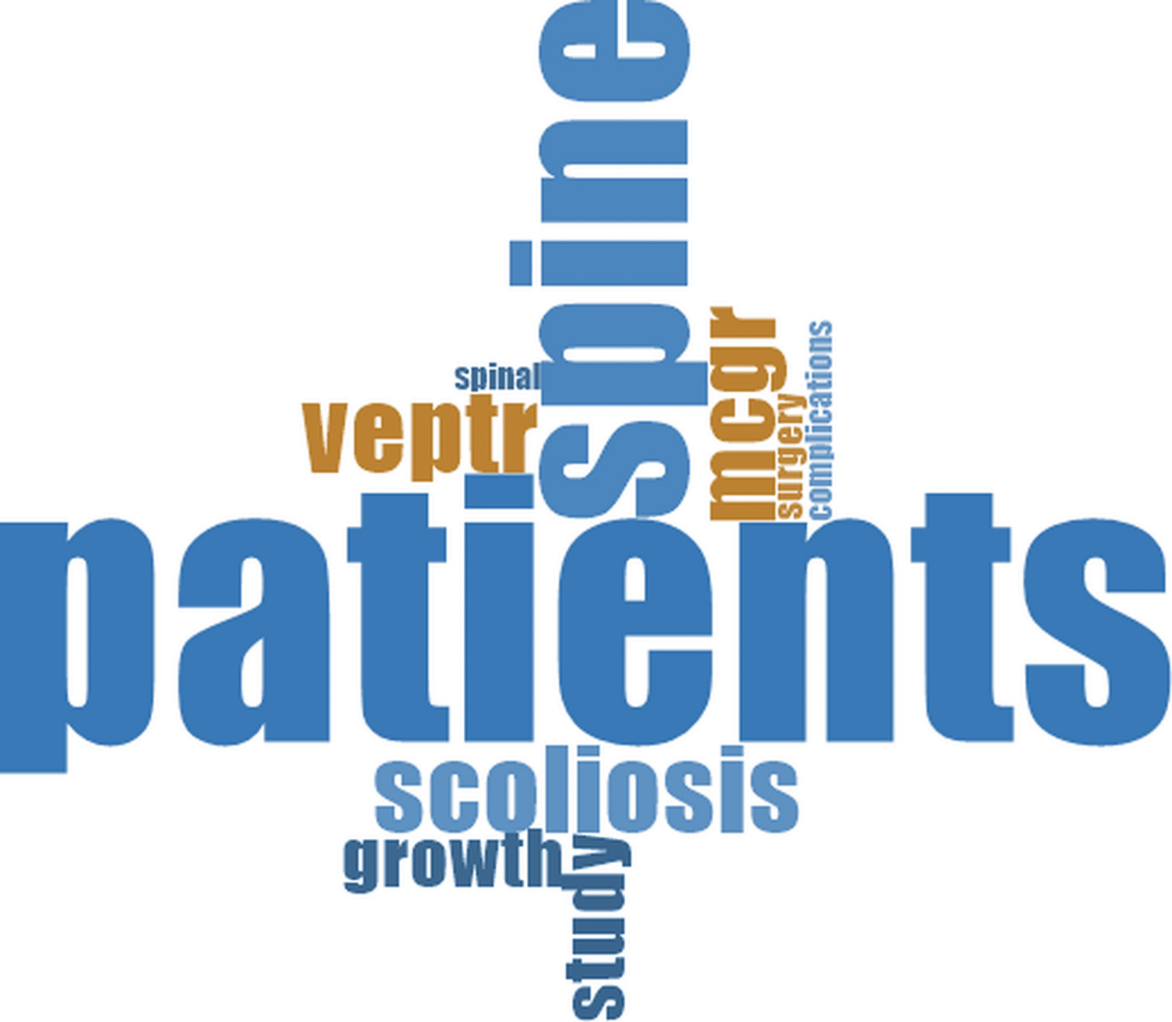
Word cloud from comparative studies between magnetically controlled growing rods (MCGR) and vertical expandable prosthetic titanium rib (VEPTR) devices

In the pairwise comparison groups, the word clouds showed recurring terms such as *patients*, *scoliosis*, *spine*, and the names of the techniques, together with repeated mentions of *surgery* and *complications* (Figures 4-6).

**Figure 7:**
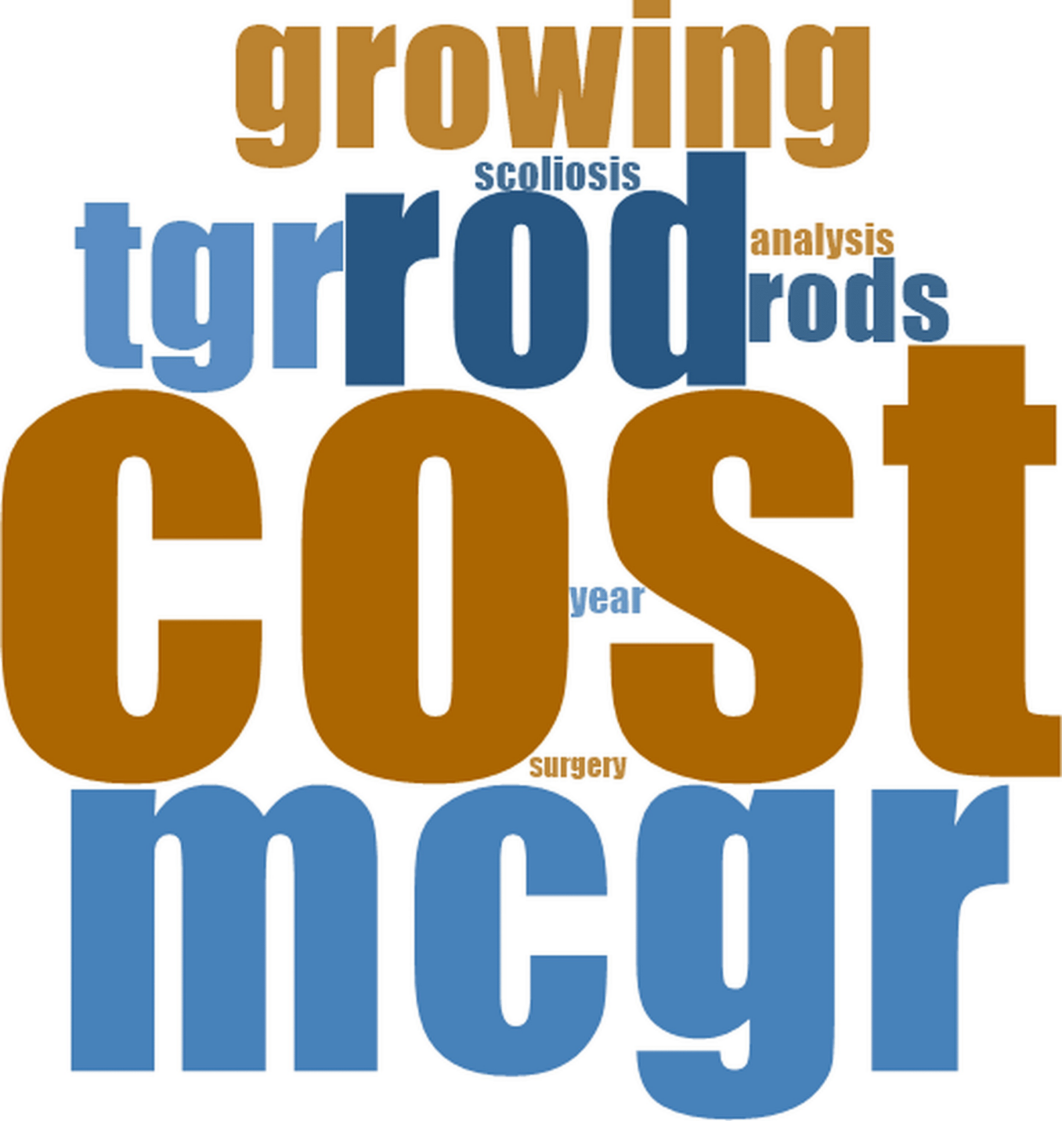
Word cloud generated from the qualitative content analysis of cost-related studies

In the economic evaluations, the term *cost* appeared prominently, accompanied by the names of the techniques and by words such as *analysis* and *surgery* (Figure 7).

Comparison Between Groups and Visual Synthesis

When comparing the different analytical groups, the word clouds showed variations in the terms that appeared most frequently. In primary comparative studies, recurrent words included patients, scoliosis, the names of the distraction-based techniques, and terms related to surgery and complications. Systematic reviews and the HTA report displayed frequent occurrences of scoliosis, spine, patients, and growth (Figure 2).

In the three pairwise comparison groups, the most common terms were patients, scoliosis, spine, and the names of the evaluated techniques, together with repeated mentions of surgery and complications (Figures 4-6).

In economic evaluations, cost was the most frequent term, typically accompanied by the names of the techniques and by terms such as analysis and surgery (Figure 7).

Across all analytical groups, patients, scoliosis, and the major distraction-based techniques appeared among the most recurrent words. The word clouds for each category (Figures 2-7) display the ten most frequent terms and summarize the terminology most commonly observed within each group.

Discussion

This study applies a qualitative, software-assisted approach to the literature on EOS by examining the most frequent terms across different study groups through word-cloud visualizations. This method complements the quantitative component of the systematic review by providing an intuitive synthesis of the predominant concepts and highlighting areas that have received disproportionate or insufficient attention.

Across primary comparative studies, the most recurrent terms were related to patients, surgical techniques, and complications. These findings reflect the persistent clinical and procedural orientation described in previous EOS reviews, in which methodological heterogeneity and limited robustness of the evidence have repeatedly been highlighted [43,45]. Systematic reviews and the HTA report displayed a similar pattern, with terminology centered on scoliosis, spine, growth, and distraction-based devices, and with comparatively little explicit reference to methodological constructs. This convergence suggests that secondary studies often reproduce the clinical focus of the available primary evidence rather than expanding the analytical perspective.

Economic evaluations, in contrast, consistently emphasized concepts related to cost and economic comparison, broadening the interpretative framework of EOS research to include resource use and efficiency. This is particularly relevant for MCGR, whose higher upfront cost has been examined in relation to potential reductions in reoperations and overall procedural burden [52,54,58].

Software-assisted content analysis tools such as NVivo have been effectively used in other biomedical fields to support transparent coding, enhance reproducibility, and facilitate the identification of thematic patterns in large textual datasets [59,60]. In EOS, this approach enabled rapid identification of domains that dominate current publications, namely surgical techniques and complications, while also revealing underexplored but clinically essential outcomes such as pulmonary function and health-related quality of life. These findings echo previous calls for broader and more patient-centered outcome assessment in this population [28,61].

Future multicenter research should incorporate robust study designs and systematically include outcomes of high clinical relevance, such as respiratory function, long-term quality of life, and cost-effectiveness. Strengthening these areas is essential to fully capture the real-world impact of distraction-based techniques and provide evidence that supports informed clinical and policy decision-making.

Limitations and Strengths

This study has several limitations. First, the qualitative analysis was exploratory and based on word-cloud visualizations, which summarize how often terms appear but do not provide numerical frequency tables or statistical comparisons between groups. For this reason, the findings must be interpreted descriptively. Second, word clouds do not show how terms are used in context, which limits the depth of interpretation. Third, the study groups were predefined according to study design, and this categorization may influence the patterns observed. Finally, because the analysis relies on the terminology used in published articles, the results reflect variability in how outcomes and concepts are reported across studies.

Despite these limitations, the study also has important strengths. It applies a transparent and reproducible approach that helps synthesize a large and heterogeneous body of EOS literature. The qualitative analysis allowed the rapid identification of which aspects are most frequently emphasized, mainly surgical techniques and complications, and which remain underrepresented, such as pulmonary function and quality of life. This broader view complements traditional systematic reviews and helps identify research gaps that are highly relevant for guiding future studies.

## Conclusions

Qualitative analysis through word clouds provides a synthetic and complementary perspective for exploring thematic patterns in the literature on distraction-based techniques for EOS. This approach enabled a clear distinction between the focus of primary and comparative studies, centered on patients, surgical techniques, and complications, and that of economic evaluations, in which cost considerations predominated.

Although the overall certainty of the available evidence remains limited, the use of software-assisted qualitative tools adds value to the systematic review process by facilitating the detection of research trends and gaps that may not be apparent through quantitative synthesis alone. This approach may serve as a foundation for future multicenter studies with greater methodological rigor and a broader scope. Particular attention should be given to underexplored domains such as quality of life, respiratory function, and cost-effectiveness, which are essential to fully capture the burden of EOS and the real-world impact of distraction-based techniques on patients’ lives.
